# Longitudinal changes and variation in human DNA methylation analysed with the Illumina MethylationEPIC BeadChip assay and their implications on forensic age prediction

**DOI:** 10.1038/s41598-023-49064-7

**Published:** 2023-12-08

**Authors:** Mie Rath Refn, Mikkel Meyer Andersen, Marie-Louise Kampmann, Jacob Tfelt-Hansen, Erik Sørensen, Margit Hørup Larsen, Niels Morling, Claus Børsting, Vania Pereira

**Affiliations:** 1 Section of Forensic Genetics, Department of Forensic Medicine, Faculty of Health and Medical Sciences, University of Copenhagen, 2100 Copenhagen, Denmark; 2 The Department of Mathematical Sciences, Aalborg University, 9220 Aalborg, Denmark; 3 The Department of Cardiology, The Heart Centre, Copenhagen University Hospital, Rigshospitalet, 2100 Copenhagen, Denmark; 4 Department of Clinical Immunology, Copenhagen University Hospital, Rigshospitalet, 2100 Copenhagen, Denmark

**Keywords:** Epigenetics, Ageing

## Abstract

DNA methylation, a pivotal epigenetic modification, plays a crucial role in regulating gene expression and is known to undergo dynamic changes with age. The present study investigated epigenome-wide methylation profiles in 64 individuals over two time points, 15 years apart, using the Illumina EPIC850k arrays. A mixed-effects model identified 2821 age-associated differentially methylated CpG positions (aDMPs) with a median rate of change of 0.18% per year, consistent with a 10–15% change during a human lifespan. Significant variation in the baseline DNA methylation levels between individuals of similar ages as well as inconsistent direction of change with time across individuals were observed for all the aDMPs. Twenty-three of the 2821 aDMPs were previously incorporated into forensic age prediction models. These markers displayed larger changes in DNA methylation with age compared to all the aDMPs and less variation among individuals. Nevertheless, the forensic aDMPs also showed inter-individual variations in the direction of DNA methylation changes. Only cg16867657 in *ELOVL2* exhibited a uniform direction of the age-related change among the investigated individuals, which supports the current knowledge that CpG sites in *ELOVL2* are the best markers for age prediction.

## Introduction

DNA methylation plays an important role in human aging^1^ and is associated with age-related diseases^2,3^. DNA methylation of the 5th carbons of cytosine residues, followed by guanines, is the most common epigenetic modification in the genome. The level of DNA methylation at some of these CpG sites can serve as a reliable biomarker for estimating an individual's chronological age and may provide insights into the aging process. This has proven a valuable tool in forensic genetics, population studies, and age-related disease research^4–7^. In forensic investigations, knowing the age of a potential perpetrator can help establish timelines and narrow down the pool of individuals that may be interesting for a police investigation^8^.

The knowledge of the relationship between DNA methylation and age mainly comes from epigenome-wide association studies (EWAS) using array technologies. These technologies were crucial for identifying CpGs with age-related methylation levels and establishing publicly available databases with epigenome-wide DNA methylation data^9^. These studies have primarily been cross-sectional, assessing the DNA methylation levels at specific CpG sites at a single point in time^10–20^, whereas longitudinal studies of methylation levels were rare^21–23^. Longitudinal studies are valuable, as they may reveal the changes in DNA methylation in individuals over time and address individual variability. Individual variability can influence the performance of age prediction models, especially when methylation levels and the direction in which they change over time are not uniform for all individuals. These effects cannot be captured with a cross-sectional approach.

EWAS were most often conducted using the Illumina Infinium HumanMethylation450K BeadChip array (450k array), while the larger Illumina Infinium MethylationEPIC BeadChip (EPIC850k array) was used in fewer studies^24^. Compared to the previous versions, the EPIC850k array represents significant improvements in the number of probes (> 850.000) and with the inclusion of probes covering regulatory elements^25^. Additionally, distal regulatory elements were not covered by the 450 k array.

Recent studies have investigated cross-sectional changes in DNA methylation correlated with age using data derived from the EPIC850k array^14,26,27^. To our knowledge, the only longitudinal study on age using the EPIC850k array is Pérez et al.^28^, where changes in DNA methylation in young individuals (birth–10 years old) were investigated.

Here, we aimed to investigate age-related changes and variation in DNA methylation using data from the EPIC850k array in a longitudinal study design with two blood samples from 64 adult blood donors collected approximately 15 years apart.

## Results and discussion

In this study, genome-wide methylation profiles of 128 blood samples were examined. Two blood DNA methylation profiles from 64 individuals were obtained using the Illumina EPIC850k array. Two blood DNA methylation profiles were generated from samples collected in 2007 and 2021, respectively. All samples passed the SeSAMe quality control. A total of 1459 probes were removed due to poor quality. Together with the default masking of 105,545 probes by SeSAMe, this resulted in 626,514 CpG sites eligible for analysis after pre-processing. Using the methylation status of these sites, we examined each CpG for linear association and inter-individual variation with age.

### Longitudinal EWAS on age

**Figure 1:**
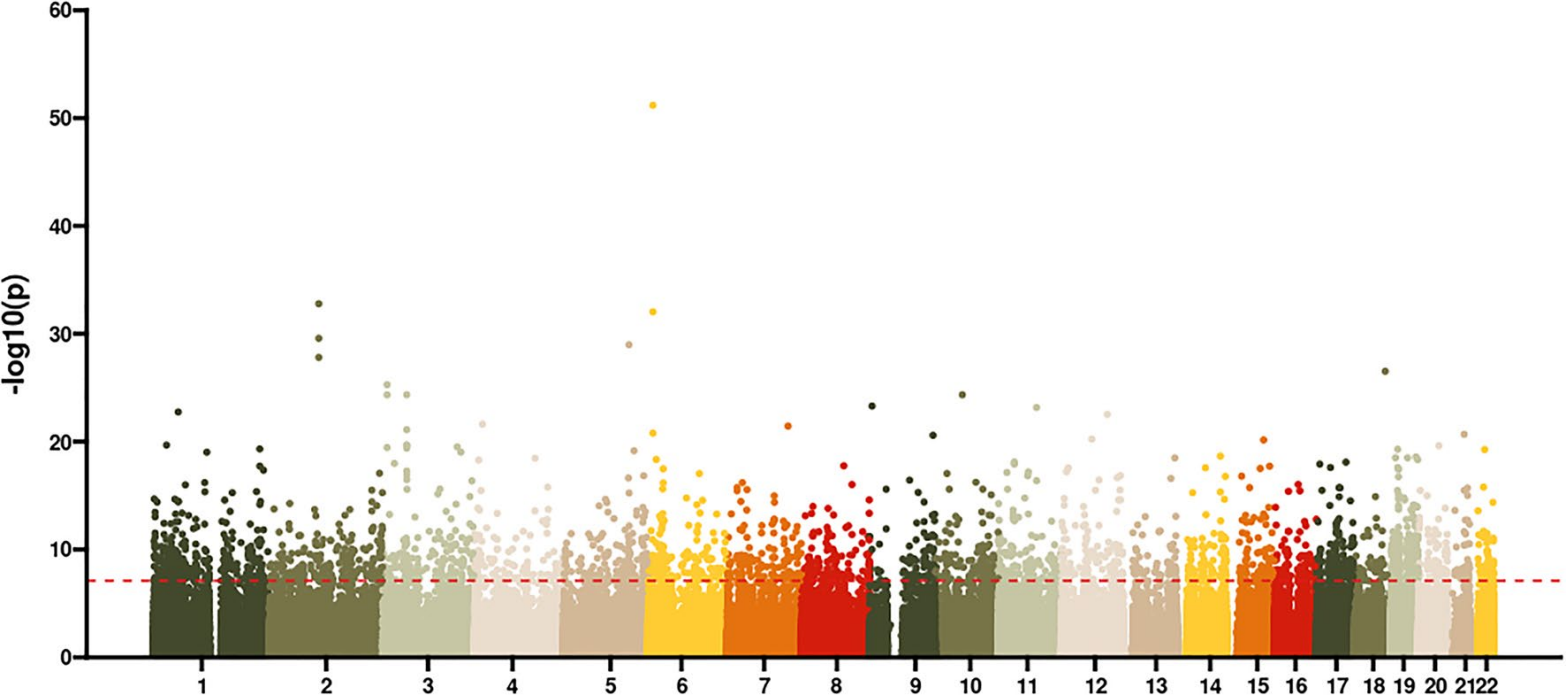
Longitudinal EWAS on age. Manhattan plot of *p *values from the mixed-effects model investigating the correlation between DNA methylation levels and chronological age. The red dashed line indicates the significance level (*p *values < 0.05) after Bonferroni correction.

Using a mixed-effects model allowing for a random intercept between individuals, a total of 2821 age-associated differentially methylated CpG positions (aDMP) were identified after correcting for multiple testing (Bonferroni corrected *p value* < 0.05), which accounted for 0.45% of the investigated probes (Fig. 1, Supplementary Table S1). Of these, 1953 were previously reported as being associated with chronological age^29^. A total of 1121 CpGs showed decreased levels of DNA methylation (hypomethylation), while 1700 CpGs showed increased levels of DNA methylation (hypermethylation) over time. With sex included as a fixed factor in the mixed-effects model, 108 CpGs were statistically significantly associated with sex (Bonferroni corrected *p value* < 0.05), three of which were also among the 2821 aDMPs. These included cg22287711 in the gene *NRG2,* cg08035323, and cg17077610. The association with sex for these three aDMPs resulted in higher methylation in females than males (Supplementary Fig. S1). Changes in DNA methylation with age can also reflect a gradual change in the cellular composition of the blood^19^. Indeed, the cellular composition of the blood samples differed between age groups (Supplementary Fig. S2). While acknowledging the potential influence of changes in the cellular composition of blood on DNA methylation patterns with age, this study aimed to capture the natural variability in DNA methylation associated with aging, including potential contributions from alterations in cell types. For this reason, neither sex nor cell composition were included in the further analysis which was restricted to a mixed-effects model that only included age as a fixed effect. This approach allows us to focus specifically on changes in DNA methylation patterns associated with age, without introducing potential confounding factors.

Of the 626,514 CpG sites investigated in this study, 291,174 were EPIC850k array extension probes. Among these, 983 were identified as aDMPs, representing 0.34% of the newly added extension probes. From the 983 CpGs, 715 were hypomethylated, and 268 were hypermethylated with age. Compared to the 335,341 probes from the previous version of the chip that are also present on the EPIC850k array, the DNA methylation detected by the extension probes was less correlated with chronological age (0.55% vs. 0.34%, *p value* > 0.05) and tended to lose methylation over time.

### Genomic region distribution

**Figure 2:**
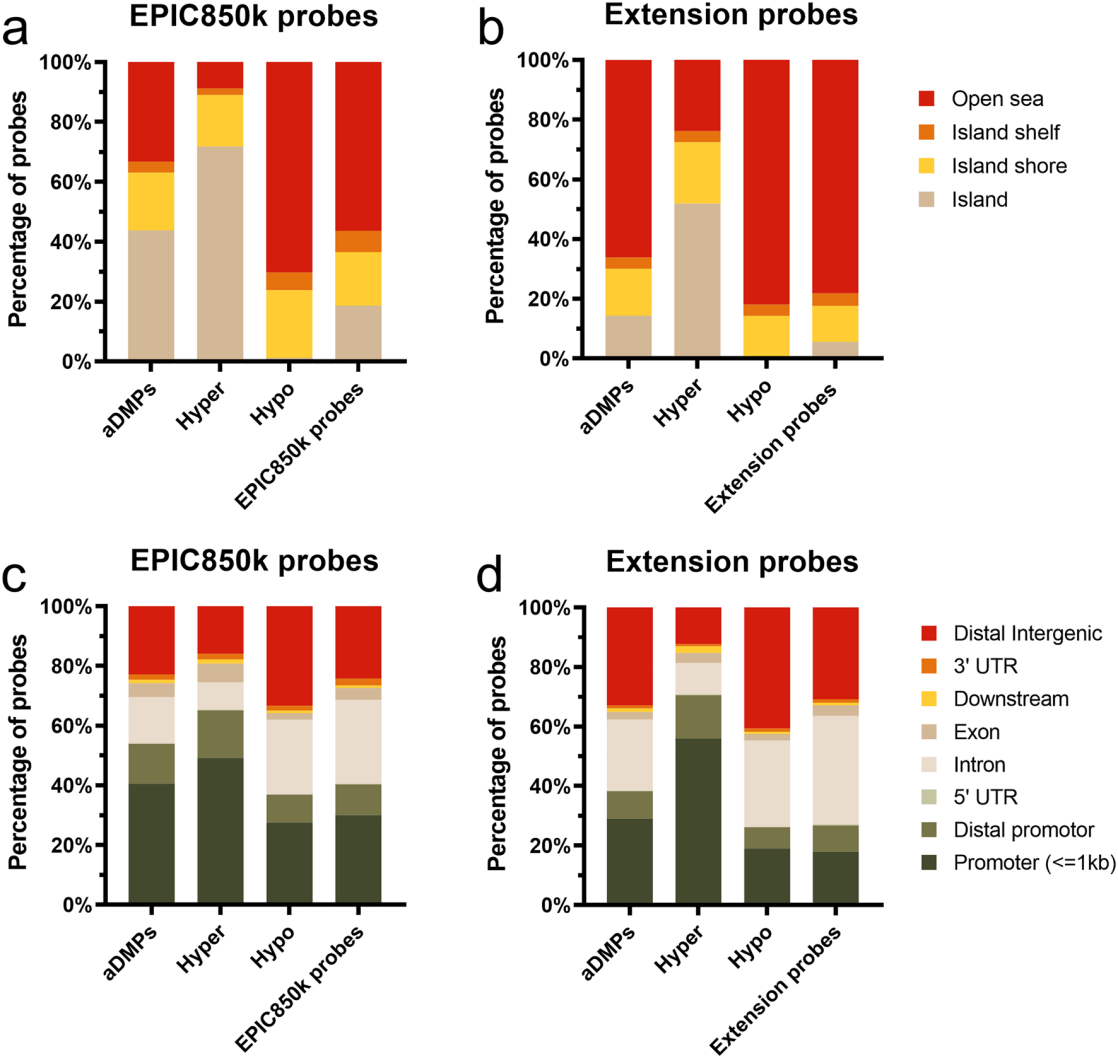
Genomic region distribution of aDMPs. Stacked bar plots of the distribution of aDMPs and positions covered by probes on the array in relation to CpG island, shelf, shores, and open sea (**a–b**) and gene-related location (**c–d**).

The genomic positions in the CpG islands of the 2821 aDMPs were examined. CpG islands are characterized by a high frequency of CpGs and are often located close to the transcription start site of genes within promoter regions^30^. CpG shores and shelves flank the CpG island and have lower CpG density than CpG island. However, they still contain far more CpGs than most of the remaining genome, which is typically poor in CpGs^30^. CpGs with increasing methylation over time were mainly located in CpG islands and less in open sea regions, while the opposite was true for hypomethylated CpGs (Fig. 2a). Furthermore, the hypermethylated aDMPs were more often located in promoter regions than hypomethylated CpGs, which were more often located in distal intergenic regions (Fig. 2c). These findings agreed with previous studies investigating age-related changes in DNA methylation^10,13,17,31^ and applied to the 450k probes as well as to the EPIC850k extension probes.

When restricting the analysis to the EPIC850k extension probes that are not included in the 450K array, it was observed that these probes were primarily located in open sea regions, consistent with their coverage of distal regulatory elements and intronic regions^25^ (Fig. 2b,d). Despite this, the aDMPs from the extension probes did not occur at these regions more frequently than the background extension probes (Fig. 2d), indicating that age-correlated changes in DNA methylation occurred less frequently at distal intergenic regions and introns than in the remaining genome offering an explanation for why a larger proportion of the 450k probes correlated with age compared to the extension EPIC probes.

### Changes in DNA methylation levels over time within and among individuals

**Figure 3:**
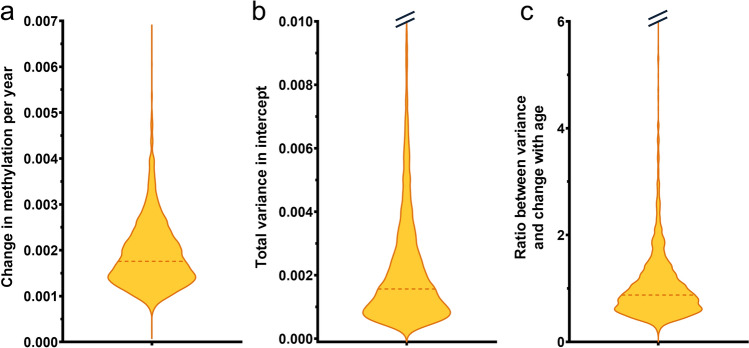
DNA methylation change and variation among individuals for the 2821 aDMPs. Violin plots of (**a**) the rate of change in DNA methylation levels within individuals per year for the 2821 aDMPs assessed by the age estimate from the mixed-effects model, (**b**) the total variance in the intercept between individuals, and (**c**) the ratio between the total variance and rate of change in DNA methylation. The horizontal dashed lines indicate the median values.

The rate of change in DNA methylation levels of the 2821 aDMPs within individuals over time was assessed by the age estimate from the mixed-effects model. This number represented the slope of the mixed effects model and thus represents the population-averaged change in DNA methylation per year. The median rate of change was 0.0018, corresponding to a change of 0.18% per year (Fig. 3a). The highest frequency of aDMPs was found at a rate of change around 0.14% per year (represented as the widest point on the violin plot in Fig. 3a). Assuming a linear correlation between DNA methylation and age, this would correspond to a range of 10–15% change over a human lifespan (~ 80 years).

The mixed-effects model was also used to investigate the association between DNA methylation levels and increased age among all individuals. Allowing for a random intercept between the individuals and assuming a consistent change in the DNA methylation levels over time provided the opportunity to investigate the inter-individual variability and estimate the variation in the intercept between the individuals. Inter-individual variation was found for all the identified aDMPs. Generally, the variance in the intercept between individuals was low, with a median variance of 0.16% (Fig. 3b).

As the rate of change in DNA methylation patterns within individuals over time was also found to be low, the variation was investigated in relation to the rate of change by taking the ratio between the age estimate and the total variance for each of the aDMPs. A ratio below 1 indicated that the variation between individuals was lower than the change in DNA methylation levels per year, whereas a ratio above 1 indicated the opposite. The ratio values ranged from 0.17 to 12.3. They had a median of approximately 0.9 (Fig. 3c), demonstrating that many of the aDMPs showed variations among individuals as large as the changes in DNA methylation levels over time. This indicated that the 2821 aDMPs showed consistency in the rate of change in DNA methylation over time, while there were significant variations in the baseline DNA methylation levels between individuals of similar ages.

As a consistent change in DNA methylation was assumed, the variation in the rate of age-related DNA methylation among individuals could not be assessed with the mixed-effects model. One possibility to address the variation would be to use a mixed-effects model allowing for random slope between individuals, where different correlations of several measurements from the same subject are accepted. However, more than two measurements at different time points would be required for this investigation. Nevertheless, it is possible to investigate the direction of the change with the information on the two measurements for each individual over the 15 years, indicating the individual changes of the DNA methylation levels with age. This type of analysis is impossible to investigate in a cross-sectional study and enables a more comprehensive understanding of the changes in DNA methylation levels with age.

The direction of the change was not consistent among all individuals except for two of the aDMPs (Supplementary Table S1). The exceptions were cg00303541 in *GRM2* and cg16867657 in *ELOVL2*, where the DNA methylation levels followed the same trend for all individuals. The fact that most aDMPs presented varying directions in the change of DNA methylation levels over time affects the age estimates in the mixed-effects model negatively, possibly explaining the lower R^2^-value observed in this study than previous studies on DNA methylation and age. On average, individuals presented opposite trends in the change in DNA methylation with age in 20% of the aDMPs. A few individuals presented opposite trends for around half of the aDMPs indicating that these individuals exhibited DNA methylation patterns that markedly differ from the majority. No correlation between the age of the individual and the number of aDMPs with opposite DNA methylation patterns was observed, and the sex of the individual did not affect the observations (data not shown). Taken together and considering that all the aDMPs showed variations in the intercept between the individuals, this result indicated that factors other than age may affect the DNA methylation levels of these CpG sites.

Inter-chip variation is a well-known source of technical variation in DNA methylation data from large-scale microarray studies and cannot always be corrected with inter-array normalization techniques^32^. It is possible to assume that differences among chips cause some variation. However, high reproducibility rates were reported between chips processed on different days^33^, and technical variation effects would most likely not be strong enough to produce the observed differences. On the other hand, inter-individual differences in DNA methylation changes over time have been shown to be associated with individual characteristics unrelated to age, such as sex, disease status, lifestyle, and ancestry^4,34–37^. Notably, sex did not seem to affect the differences in DNA methylation levels for most aDMPs. The lack of information for the blood donors beyond that of sex and age limited the exploration of how other factors might contribute to the observed variation. It is important to acknowledge that the 64 individuals included in this study were blood donors, a group generally presumed to be healthy. However, inter-individual variations in health status, genetic makeup, or lifestyle that could influence on DNA methylation may be present. Studies including a more comprehensive set of participant information could provide a more nuanced understanding of the interplay between health-related factors and DNA methylation dynamics in this specific population.

### Forensic age prediction

In forensics, the prediction of chronological age serves several important purposes, and numerous age prediction models based on changes in DNA methylation levels with age directed specifically for forensic investigations have been proposed^8^. These forensic age prediction models target a small number of CpG sites using targeted approaches for detection of DNA methylation such as pyrosequencing or massive parallel sequencing. Usually, the CpG sites were first discovered to be correlated with chronological age using array-based methods and thus some overlap in CpGs is expected between the EPIC850k array and these models. However, due to the quantitative nature of DNA methylation, some differences in DNA methylation levels between the BeadChip arrays and targeted approaches are expected, although, the main trends should be similar.

Of the 626,514 investigated CpG sites in this study, 34 were incorporated into forensic age prediction models developed for blood samples. Of these, 23 were among the 2821 identified aDMPs (forensic aDMPs), while the remaining 11 CpG sites failed to show an association with chronological age in this study. The 23 forensic aDMPs are listed in Table 1, and scatter plots of the association between chronological age and DNA methylation levels can be found in Supplementary Fig. S3.

**Table 1:** Characteristics of the 23 forensic aDMPs in blood.

CpG ID	Associated gene	GRCh38 chromo-somal position	R^2^	Age pattern	Age estimate^1^	Ratio^2^	Relation to CpG island	Genomic region	Probe type	Prediction models^3^
cg01511567	*SSRP1*	chr11:57336157	0.19	Hypomethylation	− 0.0014	0.85	Shore	Promoter (< = 1 kb)	450 k	1^38^
cg02872426	*DDO*	chr6:110415569	0.22	Hypomethylation	− 0.0048	2.35	Open sea	Promoter (< = 1 kb)	450 k	1^39^
cg04084157	*VGF*	chr7:101165768	0.35	Hypermethylation	0.0010	0.24	Island	Promoter (< = 1 kb)	450 k	1^38^
cg04528819	*KLF14*	chr7:130733488	0.22	Hypermethylation	0.0013	0.65	Island	Promoter (< = 1 kb)	450 k	2^38,40^
cg06493994	*SCGN*	chr6:25652374	0.35	Hypermethylation	0.0016	0.40	Island	Promoter (< = 1 kb)	450 k	2^38^
cg06639320	*FHL2*	chr2:105399282	0.70	Hypermethylation	0.0036	0.22	Island	Promoter (< = 1 kb)	450 k	8^6,40–48^
cg06784991	*ZYG11A*	chr1:52843096	0.55	Hypermethylation	0.0039	0.42	Island	Promoter (< = 1 kb)	450 k	1^39^
cg06874016	*NKIRAS2*	chr17:42025397	0.22	Hypomethylation	− 0.0010	0.51	Open sea	Distal promotor	450 k	1^39^
cg07082267	*No gene*	chr16:85395429	0.25	Hypomethylation	− 0.0014	0.59	Open sea	Distal Intergenic	450 k	3^43,48,49^
cg07553761	*TRIM59*	chr3:160450189	0.48	Hypermethylation	0.0035	0.52	Island	Promoter (< = 1 kb)	450 k	8^39–47^
cg08262002	*LDB2*	chr4:16573700	0.45	Hypomethylation	− 0.0028	0.46	Open sea	Intron	450 k	1^6^
cg09809672	*EDARADD*	chr1:236394382	0.33	Hypomethylation	− 0.0028	0.78	Shore	Promoter (< = 1 kb)	450 k	5^6,46,52–54^
cg13108341	*DNAH9*	chr17:11705394	0.33	Hypomethylation	− 0.0067	1.82	Open sea	Intron	EPIC850k	1^55^
cg13552692	*CCDC102B*	chr18:68722210	0.54	Hypomethylation	− 0.0036	0.42	Open sea	Intron	EPIC850k	2^49,56^
cg16054275	*F5*	chr1:169586784	0.36	Hypomethylation	− 0.0020	0.47	Open sea	Promoter (< = 1 kb)	450 k	1^39^
cg16867657	*ELOVL2*	chr6:11044644	0.83	Hypermethylation	0.0061	0.17	Island	Promoter (< = 1 kb)	450 k	7^39,54,55,57–59^
cg17268658	*FHL2*	chr2:105399288	0.62	Hypermethylation	0.0029	0.24	Island	Promoter (< = 1 kb)	EPIC850k	3^60,53,61^
cg17372101	*CNTNAP2*	chr7:147803630	0.26	Hypomethylation	− 0.0019	0.75	Open sea	Intron	450 k	1^6^
cg21572722	*ELOVL2*	chr6:11044661	0.67	Hypermethylation	0.0029	0.19	Island	Promoter (< = 1 kb)	450 k	4^6,43,48,62^
cg22736354	*NHLRC1*	chr6:18122488	0.46	Hypermethylation	0.0021	0.34	Island	Promoter (< = 1 kb)	450 k	1^38^
cg22796704	*ARHGAP22*	chr10:48465491	0.27	Hypomethylation	− 0.0025	0.91	Shore	Intron	450 k	1^6^
cg24724428	*ELOVL2*	chr6:11044655	0.45	Hypermethylation	0.0047	0.78	Island	Promoter (< = 1 kb)	450 k	2^6,62^
cg25410668	RPA2	chr1:27915066	0.43	Hypermethylation	0.0025	0.44	Shore	Promoter (< = 1 kb)	450 k	1^39^

^1^Slope of the mixed effects model.

^2^Ratio between the age estimate and the total variance.

^3^Number of forensic age prediction models that contains the CpG site.

Generally, CpG sites with high correlations between DNA methylation levels and chronological age are good age predictors. However, for age prediction in a forensic context, it is also important that the level of DNA methylation show large changes with age^6^. Samples from crime scenes often contain low amounts of DNA. The quantitative nature of DNA methylation presents a considerable challenge when the amount of input DNA is low because the accuracy of the quantified DNA methylation level can be affected by the number of cells on which the quantification is based^63^. CpG sites showing large changes in DNA methylation with age could potentially compensate for inaccuracies during the quantification of DNA methylation. It has been suggested that the change in DNA methylation with age should be higher than 70% during the human lifespan for a CpG to be a good candidate for forensic age prediction^6^. However, it is important to note that this threshold was proposed for a cross-sectional study design. The age estimate from the mixed-effects model used to assess the rate of change in DNA methylation with age in this study considers the variance between individuals. Therefore, the age estimate reflects the population-averaged value, which includes those individuals who did not follow the same trend in the mixed-effects model. As a result, the rate of change in this study is expected to be lower than in cross-sectional studies.

The median rate of change in DNA methylation per year was 0.28% for the 23 markers previously included in forensic age prediction models, which was higher than that of the remaining aDMPs (median = 0.18%). This corresponds to a change in DNA methylation of approximately 20% during a life span of 80 years. The largest range for all CpGs was approximately 50% change in DNA methylation during a lifetime, which was observed for three CpGs (cg13108341 associated with *DNAH9,* cg13552692 associated with *CCDC102B*, and cg16867657 associated with *ELOVL2*). Considering the study design, this range makes these CpGs good candidates for age prediction in forensic genetics.

The process of choosing the most suitable CpG sites for predicting chronological age has, until now, primarily relied on examining the correlation between age and methylation levels across various CpGs and, recently, also the range of change over the human lifespan, as discussed above^6^. However, neither of these parameters considers the inter-individual variation that, if present, may negatively impact the model's performance and interpretability. Inter-individual variation makes it challenging to generalize (and predict) the relationship between DNA methylation levels and chronological age, as a specific methylation level might not necessarily be uniform for all individuals of a specific chronological age. Therefore, besides aiming for CpG sites with large changes in DNA methylation with age, it is important to base the age prediction on CpG sites that show little or no variation between individuals. This would be represented by a low ratio between the age estimate and the total variation and uniform direction of the change in DNA methylation levels.

Investigation of the variation between individuals showed that the 23 forensic aDMPs generally exhibited less variation than the remaining aDMPs. The median ratio between the variation in intercept and the rate of change in DNA methylation levels over time was 0.5, placing most forensic aDMPs among the aDMPs with the lowest ratio. Furthermore, all except two forensic aDMPs had ratios below 1. This high ratio applied to cg02872426 in *DDO* and cg13108341 in *DNAH9*^39,55^. These CpGs exhibited some of the highest rates of change in DNA methylation with age. However, they also showed a high variation between individuals, making them poor markers for a forensic age prediction model. The lowest ratios were found for two CpGs associated with *ELOVL2* (cg16867657 and cg21572722) and two CpGs associated with *FHL2* (cg06639320 and cg17268658). These CpGs are among the most widely used CpGs in age prediction models (Table 1).

When looking at the direction of the change in DNA methylation level over the 15 years for the forensic aDMPs, the number of individuals that showed changes in the opposite direction of the main trend was lower than for the remaining aDMPs. Nevertheless, a few of the forensic aDMPs did not follow this trend. This included cg22796704 associated with *ARHGAP22,* cg01511567 associated with *SSRP1,* and cg04528819 associated with *KLF14*, where the change of direction was different from that of the majority in more than 26% of the 64 individuals. Neither of the three CpGs is frequently used to predict age^38,6,40^. However, other CpG sites associated with the *KLF14* gene have been used extensively in age prediction models^38–42,60^. Only the CpG site cg16867657 associated with *ELOVL2* showed the same direction of change with age for all individuals. This CpG site was among the CpGs with the highest rate of change with age and the site with the lowest ratio between the rate of change and the inter-individual variation. In a similar longitudinal study design, although only targeted towards *ELOVL2* and *FHL2*, Bacalini et al. observed similar trends for the changes in DNA methylation with age as for the present study^64^. The authors found no statistically significant variability in the intercept for *ELOVL2,* suggesting low inter-individual viability in the baseline DNA methylation of the region. This result aligns with the current findings, reinforcing that CpGs associated with *ELOVL2* demonstrate a remarkable stability in their methylation patterns across different individuals. Taken together, this explains why *ELOVL2* is currently the best predictor of chronological age. CpG sites associated with *ELOVL2* have consistently been included in nearly every DNA methylation-based age prediction model. Additionally, age estimation models based solely on methylation of CpG sites in *ELOVL2* successfully predict chronological age^57,65^. Further, the high correlation between DNA methylation and age for this region is conserved across various tissues^66,67^. In forensics, identifying CpG markers for chronological aging across multiple tissues would be useful.

The observation that most forensic aDMPs show notable inter-individual variations and some individuals show changes in DNA methylation with time that are opposite of the main trend causes some concern. Incorporation of these CpGs into an age prediction model could lead to considerable variation in performance.

## Conclusion

This study investigated genome-wide DNA methylation profiles of blood samples over 15 years. Using the Illumina EPIC850k array, 2821 age-associated differentially methylated CpG positions were identified. Differences in DNA methylation levels between individuals were observed for all the identified aDMPs, and, for many aDMPs, the variations between individuals were just as large as the rate of changes in DNA methylation per year, illustrating that while these CpGs show changes in DNA methylation that correlated with age, the level of DNA methylation varied considerably between individuals.

Of the investigated CpGs, 34 had previously been incorporated into forensic age prediction models, and 23 of these were identified as aDMPs in this study, while the remaining 11 failed to show age correlation. These 23 CpG sites generally showed lower variation and a higher rate of change with age compared to the remaining aDMPs. However, opposite trends in DNA methylation changes with age were observed for most forensic aDMPs. Only the CpG site cg16867657 in *ELOVL2* had a uniform change with age among the investigated individuals.

Despite the significant insights gained from this study on inter-individual variations in changes in DNA methylation patterns over time, the relatively small number of samples may impact the generalization of the findings. Further research involving larger cohorts with broader age spans and more data points is essential to validate and extend these results and completely unravel the complexities of inter-individual variations in DNA methylation patterns. Furthermore, longitudinal studies should be conducted using DNA methylation detection methods applicable to forensics genetics to fully understand the potential implications of inter-individual variations for implementation into forensic genetics.

## Materials and methods

### Study cohort

This study was conducted using fully anonymised samples from an existing biobank of archived blood samples from blood donors in The Capital Region of Denmark. All the experiments were in accordance with relevant guidelines. The biobank was approved by the Scientific Ethics Committees in Region Zealand and Region Central Denmark (ID numbers 1-10-72-95-13 and SJ-740) and registered according to the rules defined by the Danish Data Protection Agency (ID: P-2019-99). The archived samples consist of material from routine blood donations, where the donors have given written consent to use the blood samples in research projects. The study follows the policy from the National Science Ethics Committee in Denmark. All samples were fully anonymised and comply with the rules of the General Data Protection Regulation [Regulation (EU) 2016/679]. Two blood samples from each of 64 fully anonymised individuals who had donated blood repeatedly since 2007 were used, one collected in 2007 and another in 2021. The individuals were sex and age-matched. One male and one female of all ages between 18 and 49 years at the first sampling were included (Supplementary Fig. S4). The age of the individuals was provided in years and quarters. Five mL blood was drawn from a peripheral vein using a BD Vacutainer® PPT™ Plasma Preparation Tube Vacutainer containing a separation gel (BD Biosciences, Franklin Lakes, NJ, USA) and centrifuged according to the manufacturer’s protocol to separate the plasma from the cellular elements before being stored at − 20 °C.

### Sample preparation and DNA extraction

Before DNA extraction, samples were prepared by removing the plasma, leaving the cellular elements underneath the gel at the tube bottom. The samples were centrifuged upside down at 3700 g for three minutes for the cellular elements to move to the top of the tubes. The samples were stored at 4 °C until the next day. DNA was extracted using the QIAamp DNA Mini Kit (Qiagen, Hilden, Germany) using 400 µL of the sample and 100 µL Buffer AE in the elution step. Centrifugation of the lysate was performed with 8000 g to avoid clogging of the membrane. DNA quantification was performed using the Qubit dsDNA High Sensitivity (HS) Assay Kit on a Qubit 2 Fluorometer (Thermo Fisher Scientific, Waltham, MA, USA) following the manufacturer’s guidelines.

### Bisulphite conversion of genomic DNA

DNA samples were bisulphite converted using the EZ DNA Methylation™ Kit (Zymo Research, Irvine, CA, USA) following the manufacturer’s recommendations using 125 ng of gDNA as input as described by Christiansen et al.^33^. Briefly, the gDNA was denatured using Zymo M-Dilution buffer and incubated for 15 min at 37 °C. Bisulphite conversion was carried out by adding CT-conversion reagent followed by 16 cycles of 95 °C for 30 s and 50 °C for 60 min. in the thermocycler. After desulphonation and cleaning, the bisulphite treated DNA was eluted in 10 µl M-Elution Buffer.

### DNA methylation analysis

DNA methylation was measured using the Infinium MethylationEPIC Kit (Illumina, San Diego, CA, USA) according to the manufacturer’s protocol. In brief, 4 µL of bisulphite converted DNA was whole-genome amplified (WGA) before being enzymatically fragmented, precipitated, and resuspended in the hybridization buffer. The fragmented DNA was hybridized to probes attached to the BeadChips and processed through single-base primer extension. Lastly, the BeadChips were stained and imaged using the iScan™ system (Illumina). The samples were allocated onto 18 BeadChips and processed in five separate batches (four batches with four chips and one batch with two chips). The samples derived from the same individual were loaded randomly on the same chip in neighboring wells to reduce the between-chip variance. An even number of males and females were distributed on each chip. The samples from The Human Methylated & Non-Methylated (WGA) DNA Set (Zymo Research) were included as references.

### Data pre-processing

The data analysis was performed in the statistical environment R (version 4.2.1)^68^. The resulting raw Intensity Data files (.idat) from the iScan™ were imported into R and processed using the SeSAMe package (version 1.16.1)^69^ with the openSesame() pipeline that provided end-to-end processing and converted .idat files into DNA methylation levels. In brief, the processing consisted of (1) masking of 105,454 non-uniquely mapping probes, (2) channel inference for Infinium-I probes, (3) non-linear dye bias correction, (4) masking of low-quality probes (defined as probes exhibiting detection *p v*alues of ≥ 0.05) computed using out-of-band (oob) probes empirical distribution (pOOBAH), and (5) background subtraction based on normal-exponential deconvolution using oob probes (noob). Quality control was performed on the raw data using the SeSAMe QC quality metrics from the SeSAMe package (version 1.16.1)^69^ including information on sequence quality, read length distribution, GC content, overrepresented sequences, and more. This can in turn be used to assess the quality of each sample. Finally, the normalized intensity signals were converted into DNA methylation levels presented as beta(β)-values. The β-value is a continuous variable between 0 and 1, representing the proportion of methylated (M) to the total amount of methylated and unmethylated (U) CpG nucleotides, i.e., β = M/(M + U). The analysis was restricted to data from probes reacting with CpG dinucleotides on autosomes. Only data from probes with beta values for all samples were included.

### Differential DNA methylation analysis

To describe the longitudinal changes in DNA methylation in individuals over time accounting for correlation between measurements of the same individual, a linear mixed-effects model was created in R using the *lmer* function in the *lmerTest* package^70^. This type of model is useful to understand how individual differences (random effects) and overall patterns (fixed effects) contribute to the variability in the data. In this work, the model considered β-values as the response, fixed effects of age, and random intercepts for each individual, to account for the individual differences from the average baseline value. The same approach was applied including sex as a fixed effect to test sex-associated differences in DNA methylation levels. The marginal R-squared (R^2^) values were calculated using the *MuMIn* package. The *p values* obtained were adjusted for multiple testing according to the Bonferroni method.

Cell composition was estimated using the R packages *EpiDISH* (version 2.18.0) with the individuals grouped in the following age groups: < 20 years, 20–29, 30–39, 40–49, 50–59, and < 60 years. Relationship to CpG island and gene association was assigned to each probe using the Illumina EPIC annotation file with the R/Bioconductor annotation package *IlluminaHumanMethylationEPICanno.ilm10b4.hg19* (version 0.6.0). Genomic region annotation was assigned using the R/Bioconductor packages *TxDb.Hsapiens.UCSC.hg19.knownGene* (version 3.2.2) and *ChIPseeker* (version 1.24.0)^71^. Previous association with age was investigated using the EWAS Toolkit under the EWAS Atlas^29^ (available online at https://ngdc.cncb.ac.cn/ewas/atlas/index).

### Supplementary Information

Supplementary Figures.

Supplementary Tables.

## Data Availability

The datasets used during the current study is available from the corresponding author on reasonable request.
