# Supplementary material for: Longitudinal changes and variation in human DNA methylation analysed with the Illumina MethylationEPIC BeadChip assay and their implications on forensic age prediction
Source: Sci Rep. 2023 Dec 8;13:21658. doi: 10.1038/s41598-023-49064-7 (PMC10709620; doi:10.1038/s41598-023-49064-7)
Supplement: Supplementary file 1 — Supplementary Figures. [file 41598_2023_49064_MOESM1_ESM.pdf]

Supplementary figures

Longitudinal changes and variation in human DNA methylation analysed with the Illumina MethylationEPIC BeadChip assay and their implications on forensic age prediction

Mie Rath Refn, Mikkel Meyer Andersen, Marie-Louise Kampmann, Jacob Tfelt-Hansen, Erik Sørensen, Margit Hørup Larsen, Niels Morling, Claus Børsting and Vania Pereira

Supplementary Figure S1

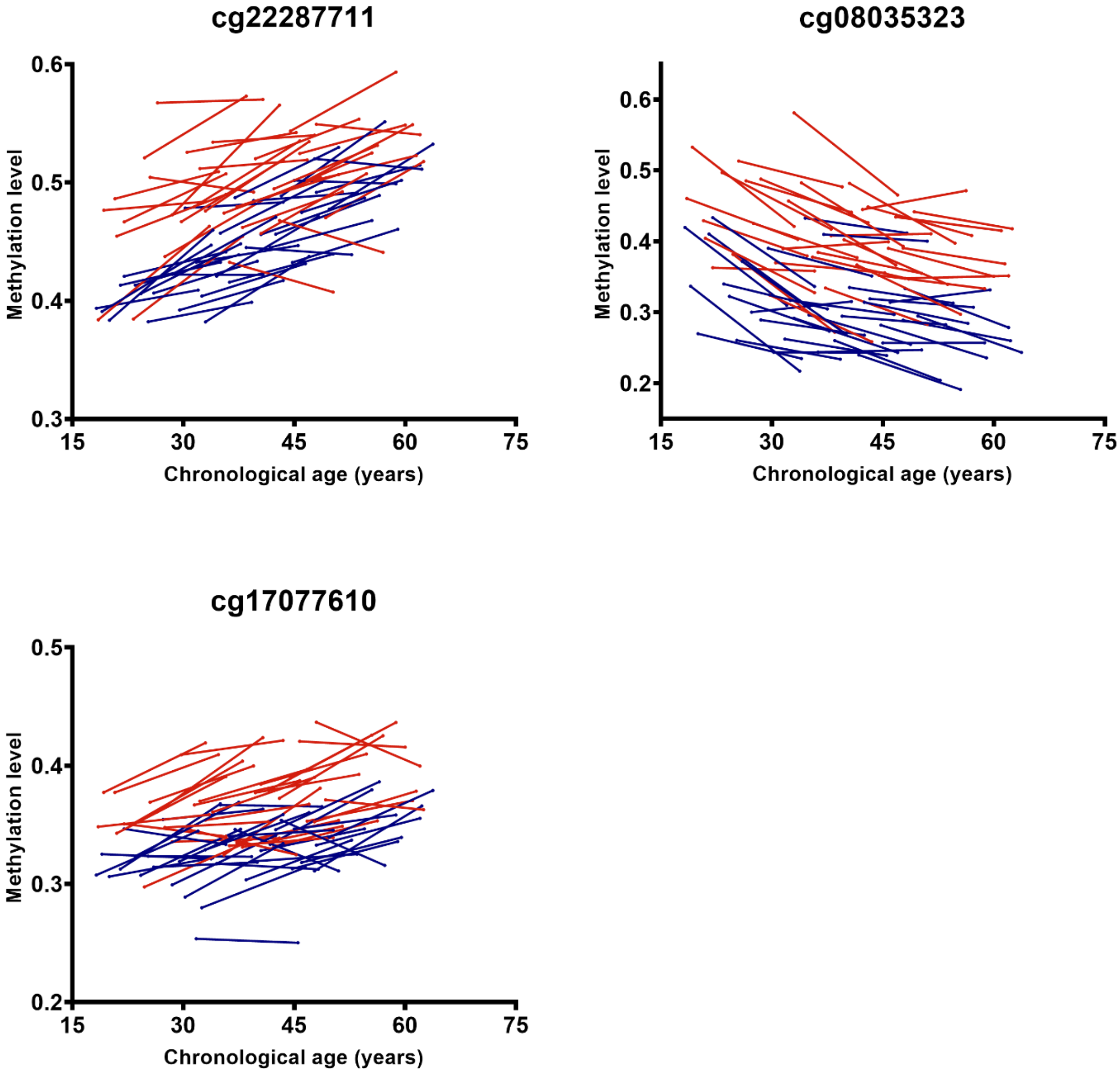

Supplementary Figure 1. Influence of sex on age-related changes in DNA methylation. Scatter plots of the methylation levels versus chronological age for the four aDMPs correlated with sex and age in the analysis. The connecting lines indicate samples from the same individual. Red lines indicate females, and blue lines indicate males.

Supplementary Figure S2

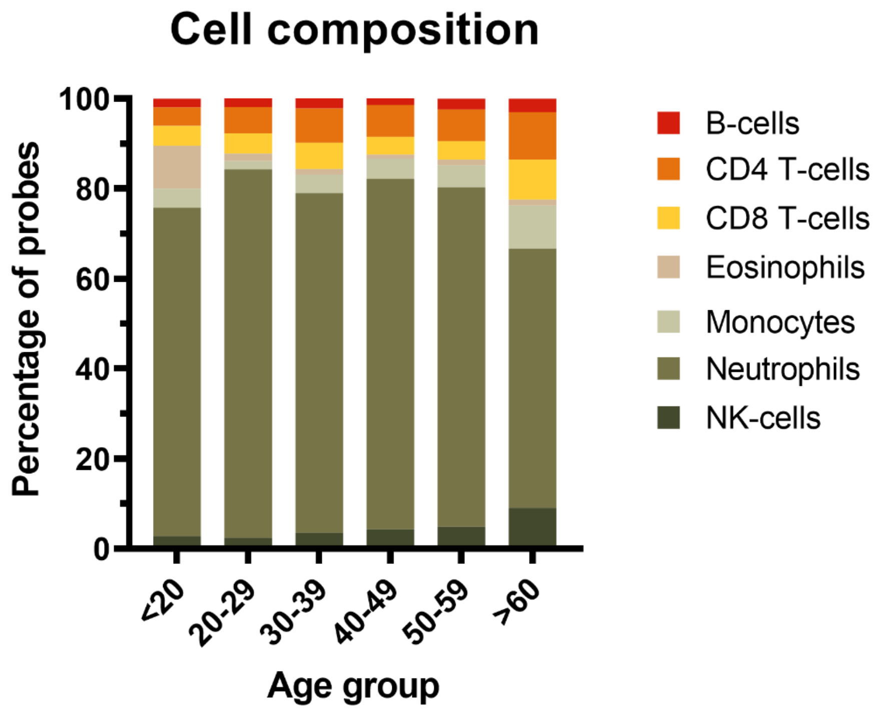

Supplementary Figure 2. Changes in cell composition with age. Stacked bar plots of the cell composition in the blood samples from the participating blood donors categorized into age bins.

Supplementary Figure S3

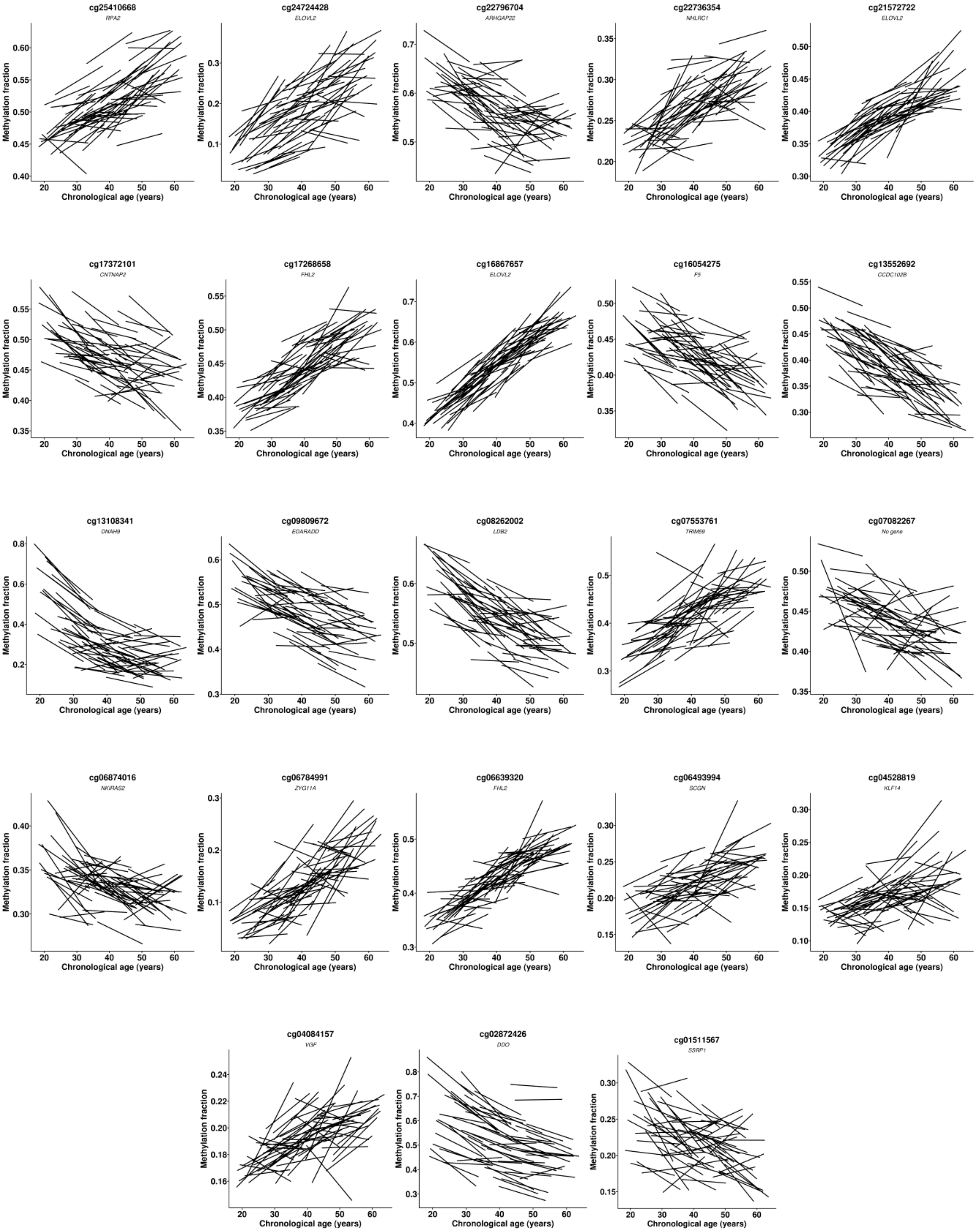

Supplementary Figure 3. Forensic aDMPs. Scatter plots of the methylation levels versus chronological age for the 23 forensic aDMPs. The connecting lines indicate samples from the same individual.

Supplementary Figure S4

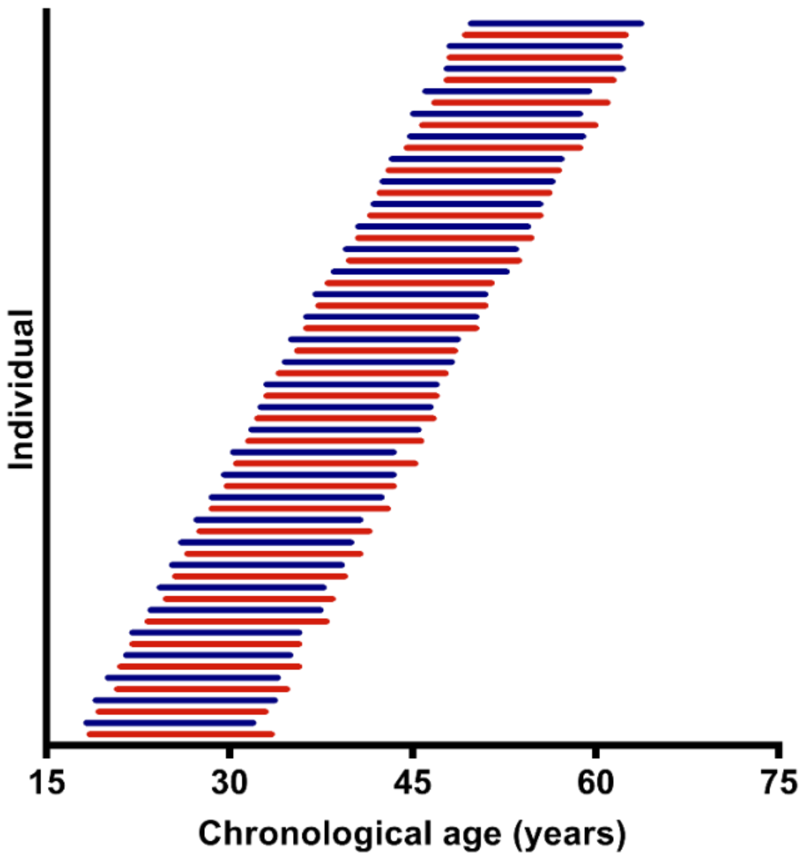

Supplementary Figure 4. Longitudinal sampling of the participating blood donors. The ends of the lines indicate the age at sampling from each of the 64 participating blood donors at the first (2007) and second (2021) blood donations. Red lines indicate female donors, and blue lines indicate male donors.
